# Cytomegalovirus infection in infants with biliary atresia in China: a multi-center investigation study

**DOI:** 10.3389/fped.2025.1577113

**Published:** 2025-06-06

**Authors:** Yilin Zhao, Xiaodan Xu, Yu Meng, Chonggao Zhou, Baijun Zheng, Xianwei Zhang, Bin Wang, Xufei Duan, Yuanmei Liu, Jiexiong Feng, Jie Zhu, Zhibo Zhang, Yajun Chen, Zhibao Lv, Yuanjun Hu, Hongxia Ren, Liuming Huang, Yingchao Li, Liang Ge, Haochuan Zhang, Xiang Liu, Wanfu Li, Qingbo Cui, Jianghua Zhan

**Affiliations:** ^1^ Department of General Surgery, Tianjin Children’s Hospital (Children’s Hospital, Tianjin University), Tianjin, China; ^2^ Graduate College, Tianjin Medical University, Tianjin, China; ^3^ Department of General Surgery, Hunan Children’s Hospital, Zhuzhou, Hunan, China; ^4^ Department of Pediatric Surgery, The Second Affiliated Hospital of Xi’an Jiaotong University, Xi’an, Shanxi, China; ^5^ Department of General Surgery, Henan Children’s Hospital, Zhengzhou, Henan, China; ^6^ Department of General Surgery, Shenzhen Children’s Hospital, Shantou, Shenzhen, China; ^7^ Department of General Surgery, Wuhan Children’s Hospital, Wuhan, Hubei, China; ^8^ Department of Pediatric Surgery, The Affiliated Hospital of Zunyi Medical University, Zunyi, Guizhou, China; ^9^ Department of Pediatric Surgery, Tongji Hospital of Tongji Medical College, Huazhong University of Science and Technology, Wuhan, Hubei, China; ^10^ Department of General Surgery, Children’s Hospital of Soochow University, Suzhou, Jiangsu, China; ^11^ Department of Neonatal Surgery/Pediatric Surgery, Shengjing Hospital of China Medical University, Shenyang, China; ^12^ Department of General Surgery, Beijing Children’s Hospital, Capital Medical University, Beijing, China; ^13^ Department of General Surgery, Children’s Hospital of Shanghai, Children’s Hospital Affiliated to Shanghai Jiao Tong University School of Medicine, Shanghai, China; ^14^ Department of Neonatal Surgery, Jinan Children’s Hospital, Jinan, Shandong, China; ^15^ Department of Neonatal Surgery, Children’s Hospital of Shanxi, Taiyuan, Shanxi, China; ^16^ Department of Pediatric Basic Surgery, Chinese Plageneral Hospital Chinese PLA Medical School, Beijing, China; ^17^ Department of Pediatric Surgery, The Second Hospital of Hebei Medical University, Shijiazhuang, Hebei, China; ^18^ Department of Pediatric Surgery, The 2nd School of Medicine. WMU/The 2nd Affiliated Hospital and Yuying Children’s Hospital of WMU, Wenzhou, Zhejiang, China; ^19^ Department of Neonatal Surgery, Anhui Provincial Children’s Hospital, Anhui, China; ^20^ Department of Pediatric Surgery, The First Affiliated Hospital of Xinjiang Medical University, Urumqi, Xinjiang, China; ^21^ Department of Pediatric Surgery, The Sixth Affiliated Hospital of Harbin Medical University, Harbin, Heilongjiang, China

**Keywords:** biliary atresia, cytomegalovirus, multicenter, diagnosis, treatment

## Abstract

**Background and objectives:**

Biliary atresia (BA) with concurrent cytomegalovirus (CMV) is a distinct subtype that is linked to a poorer prognosis. Currently, there are no standardized criteria for the diagnosis or antiviral treatment (AVT) of this condition. It has a high prevalence in China. The aim was to investigate the infection, diagnosis and treatment of CMV infection in infants with BA through a multicenter questionnaire survey conducted in China.

**Methods:**

A multicenter investigation was performed through online questionnaire survey. It investigated the diagnosis and treatment of infants with CMV-infected BA in tertiary-level pediatric centers from January 1st, 2018, to January 1st, 2020. The centers were categorized into low and high-volume groups based on number of infants with BA (≤50 or >50) and were also grouped geographically into south and north groups. Afterward, 100 cases were randomly selected from these infants for a retrospective analysis.

**Results:**

A total of 22 questionnaires were collected, and 20 were included in the analysis. The questionnaire survey encompassed 1,276 infants with type III BA. 31.3% of the infants of BA had CMV detected. According to the survey results, a large proportion of centers preferred using CMV-DNA (75.0%) and CMV-IgM (95.0%) as their preferred methods for CMV detection. In the high-volume group, more centers opted for CMV-DNA detection (100.0% vs. 66.7%) and administered AVT (87.5%). In the retrospective analysis of 100 infants with BA, 39 were found to be CMV-positive and among these, 74.4% received AVT.

**Conclusion:**

Among the 1,276 infants with BA in this cohort, 31.3% (399 cases) had concomitant CMV infection, representing a decrease compared to previous data. CMV-IgM played a crucial role in the detection of the infection. The retrospective analysis indicated that AVT had a beneficial impact on the prognosis of infants with BA who were infected with CMV.

## Highlights

• This is the first multicenter investigation conducted in China on the diagnosis and treatment of infants with BA infected with CMV.
• A total of 20 children medical centers were included in this study.
• In this investigation, the prevalence of CMV infection in infants with BA in China was approximately 31.27%.

## 1 Introduction

Biliary atresia (BA) is a destructive and obliterative cholangiopathy that occurs in infants (1). It is characterized by persistent hepatic inflammation and progressive liver fibrosis (2). The incidence of BA ranges from 1/5,000 to 1/20,000, with higher rates observed in Asian countries (1.51/10,000) (3, 4). The primary treatment for BA is Kasai portoenterostomy (KPE), which prolongs native liver survival (NLS) (5, 6). If liver fibrosis is advanced, liver transplantation (LT) is considered the definitive treatment to enhance long-term survival (7). The etiology of BA remains unclear, but it may be associated with viral infection, immune disorders, or inflammation (8–10).

Cytomegalovirus (CMV) is a hepatophilic double-stranded DNA virus that can cause isolated organ injury or multisystem involvement, including liver, nervous system, and blood system (9). Davenport (3) identified CMV-IgM positive BA as a subtype of BA. Evidence of CMV infection has been reported in 10%–38% of infants with BA in European studies, with higher rates observed in Asia, particularly in China, where rates have reached up to 60% in 2012 (5, 9, 11). There were notable differences in the detection and treatment of infants with CMV-infected BA, with various methods employed in different studies, including CMV-IgM, CMV-DNA, and CMV-pp65 for detecting CMV infection (9, 12, 13).

Several studies have suggested that CMV infection impacts the prognosis of infants with BA (9, 13, 14). A meta-analysis conducted by previous research indicated that infants with CMV-infected BA experienced worse jaundice clearance (JC) (15). Fischler's study found that antiviral treatment (AVT) improved the prognosis of infants with CMV-infected BA (14). Moreover, valganciclovir or sequential treatment with valganciclovir and ganciclovir was primarily used in Western countries, while ganciclovir was more commonly administered in China (12, 16). The dosage of treatment varied by country and center.

In conclusion, a multicenter survey is essential for guiding the treatment of infants with CMV-infected BA. A large sample of infants with CMV-infected BA in China would be beneficial for conducting such research. The aim of this study was to provide a descriptive analysis of the diagnosis and treatment of CMV-positive BA in multiple centers in China using a questionnaire, which can serve as a valuable reference for future research in this area.

## 2 Methods

### 2.1 Participant centers

A multicenter investigation was conducted using online questionnaires across 22 tertiary-level pediatric centers in China (Supplementary Table S1). This investigation was carried out through the Pediatric Surgery Multi-Center Medical Collaborative Association, which was established in China in 2015. This association comprises 40 pediatric medical centers and is dedicated to researching a wide range of pediatric diseases, including BA and megacolon. Regular in-person meetings are held annually to facilitate discussions and review the progress of ongoing projects.

This online questionnaire was directly administered to the leader of each medical team. The staff responsible for follow-up varied among centers. In some centers, a dedicated staff member was responsible for case information, while in others, a clinician took on this responsibility. Therefore, the questionnaire was completed by the individual who had knowledge of the patients' clinical data at each center, and a signature was required upon completion. This approach ensured that the data collected was both reliable and accurate.

The centers were categorized into two groups based on their caseload of eligible infants with BA from 2018 to 2020: a low-volume group (≤50 patients) and a high-volume group (>50 patients). Additionally, China was divided into northern and southern regions by the Qinling-Huaihe River, and the centers were further classified into south group and north groups according to their geographical location.

### 2.2 Questionnaires

The questionnaire was posted online and comprised 31 questions (Supplementary Material S1). The questionnaire items were initially proposed by experts during in-person meetings, with the first draft prepared by the corresponding author. The questionnaire then underwent two rounds of revisions through additional face-to-face discussions and the final version was approved by all experts. The questions were designed based on fundamental clinical knowledge, ensuring that the content was straightforward and easy to comprehend for the respondents, all of whom were practicing clinicians, which was helpful for maintain consistency throughout the questionnaire. The questionnaire was distributed at the National Pediatric Surgery Multi-center Annual Meeting, where a dedicated session was held to explain the questions and address any concerns. Each medical center was required to collect clinical data, which necessitated sufficient time for completion. Therefore, a three-month period was allocated for data collection after the questionnaire was distributed. Respondents were expected to spend at least 30 min filling out the questionnaire to ensure that clinical doctors had enough time to complete it thoroughly. After data collection, expert discussions were conducted in offline meetings to verify the accuracy of the responses before incorporating them into the final analysis. The design and implementation process of the questionnaire was illustrated in Supplementary Figure S1. Each questionnaire could be completed only once per Internet Protocol Address (IP), and a signature was required from the individual who completed it. Homogeneous questions were included to assess the quality of the responses. If the answers to these questions were contradictory, the questionnaire was deemed ineligible, and excluded from the analysis. The questionnaire was created using the Questionnaire Star website, an online platform for surveys and was sent to the leader of each medical center.

The questionnaire was divided into two parts. The first part focused on the diagnosis and treatment approaches of infants with CMV-infected BA. The diagnosis section included questions about virus detection methods, detection time and the diagnostic criteria for infants with CMV-infected BA. The second part of the questionnaire pertained to prognosis. To ensure the authenticity of the data, this section was optional due to the challenges associated with follow up; not all centers were able to complete it. A medical center could still submit the questionnaire even if the second part was not filled out. The patient's prognosis outcome (expressed as percentage) was entered directly rather than uploading of individual patients' clinical data. The prognostic indicators included JC, early cholangitis, frequent cholangitis, and NLS (Supplementary Material S1).

### 2.3 BA patients included in the questionnaire

The questionnaire included infants with BA who were diagnosed and underwent Kasai between January 1, 2018, and January 1, 2020, with follow-up until January 31, 2021. Specified criteria for inclusion in the study were established, including a confirmed diagnosis of type III BA, Kasai surgery, parental consent, and the absence of BASM or other severe malformations that could lead to mortality. Infants with CMV-positive BA was defined as meeting one of the following conditions: (1) CMV-DNA positive in the blood or urine; (2) CMV-pp65 positive in the blood or urine; (3) CMV-IgM positive in the blood; (4) serum CMV-IgG antibody titer ≥4 times the baseline; or (5) presence of CMV inclusion bodies in the urine. Infants with BA who did not meet any of the indicators were classified as CMV-negative.

Following the questionnaire investigation, 100 cases were randomly selected from the included patients for a detailed retrospective analysis of their clinical data. Comprehensive clinical information was collected, including basic patient demographics, preoperative blood routine and liver function tests, preoperative CMV testing, and prognosis outcomes.

This study was conducted in accordance with the Declaration of Helsinki. Approval for the research protocol was obtained from the Ethics Committee of Tianjin Children's Hospital (2022-SYYJCYJ-008). Informed consent was obtained from the legal guardian(s) of each patient.

### 2.4 Sample size calculation

Based on previous multicenter retrospective studies on BA in mainland China, the ratio of CMV-positive BA to CMV-negative BA is approximately 1:2. Using the JC rates from previous meta-analysis, assuming that the rate of CMV-positive BA is 50% and that of CMV-negative BA is 60%. Statistical analysis was performed using the Chi-square test with a level of 0.05 and 1—*b* = 0.9. This analysis indicated that 1,167 infants with BA should be included in the study, comprising 389 being infants with CMV-positive BA and 778 infants with CMV-negative BA. PASS 15.0 is used to calculate sample size.

### 2.5 Statistics

This study is mainly descriptive research. After grouping by region or caseload volume of people, a statistical analysis was carried out using SPSS22.0. *P* < 0.05 was considered to indicate statistical significance.

## 3 Results

### 3.1 Overview

**Figure 1:**
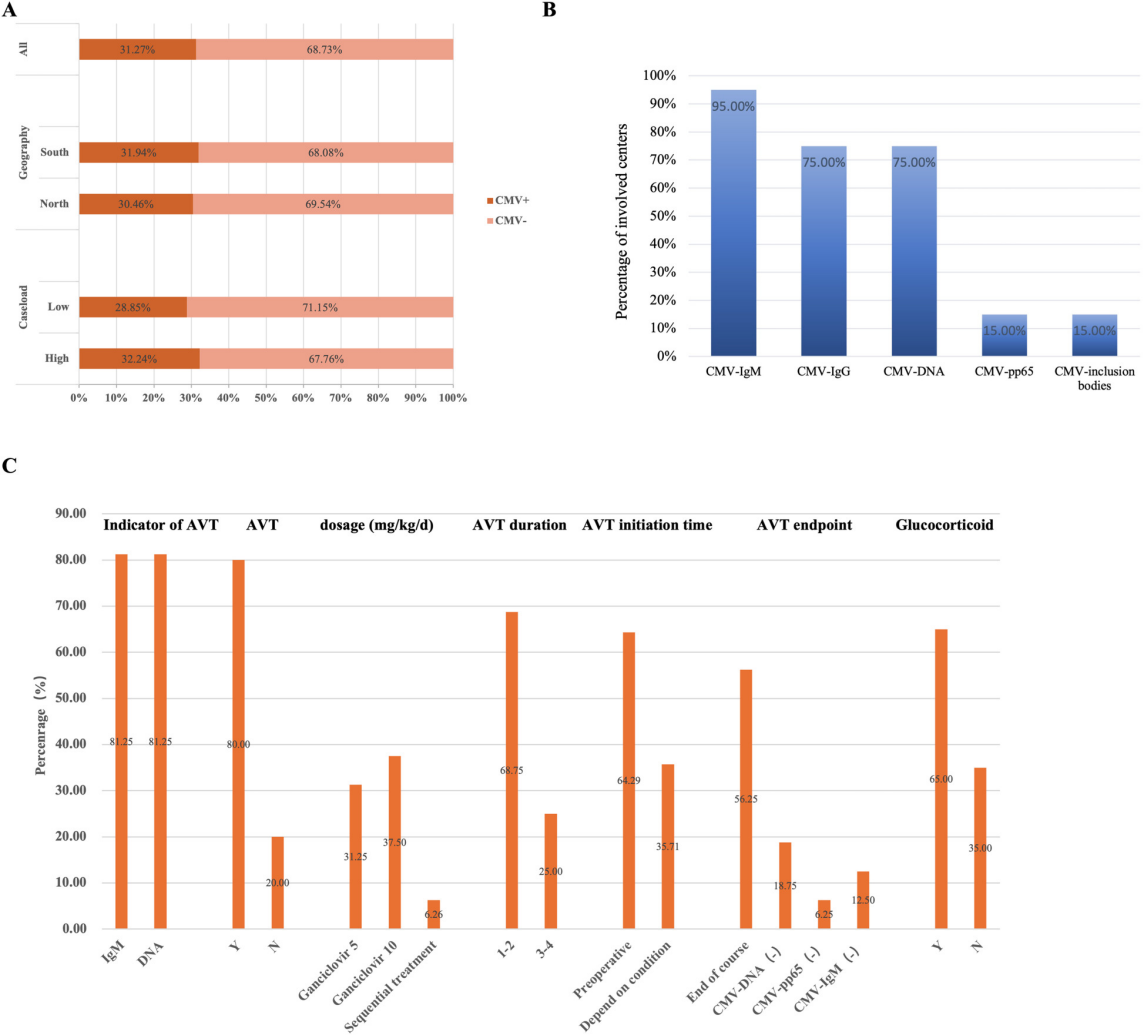
CMV infected infants with BA across 20 centers. **(A)** The CMV infection status of infants with BA in different subgroups. Among all included infants, 31.3% of infants with BA were CMV-positive, while 68.7% were CMV-negative. By region, 31.9% of infants with BA in south group were CMV-positive, compared to 30.5% of infants with BA in northern medical centers. By patient volume, 32.2% of infants with BA in the high-volume group were CMV-positive, whereas 28.9% of infants with BA in the low-volume group. **(B)** Multi-center CMV detection methods. The proportion of medical centers using various CMV detection methods among all centers surveyed. **(C)** The diagnosis and treatment of infants with CMV^+^ BA in 20 medical centers. The proportion of medical centers selecting different responses to the survey question.

A total of 22 questionnaires were collected, and of which 20 were valid, 2 questionnaires were deemed ineligible due to discrepancies in the responses to homogeneity questions. The data analyzed were obtained from 20 medical centers located across 17 provinces in China (Supplementary Table S1). A total of 1,276 infants with BA were identified through the questionnaire survey conducted across the 20 participating centers, including 399 (31.3%) infants with CMV-positive BA and 877 (68.7%) infants with CMV-negative BA (Figure 1A).

Based on the questionnaire survey, among the 20 centers, 11 centers classified in the north group which included 581 infants with BA, of whom 177 (30.5%) CMV-positive cases. Nine centers were located in the south group, accounting for 695 infants with BA, including 222 (31.9%) CMV-positive cases. According to the caseload grouping derived from the survey, 12 centers were categorized as low group, with a total of 364 infants with BA of which 105 (28.9%) were CMV-positive cases, and 8 centers were classified as high group, comprising 912 infants with BA, including 294 (32.2%) CMV-positive cases (Figure 1A).

### 3.2 Examinations and diagnosis

In the first part of the questionnaire survey, most centers performed the CMV-IgM test (19/20) and CMV-DNA test (15/20). In contrast, only a few centers tested CMV-pp65 (3/20) and CMV inclusion bodies (3/20) (Figure 1B). Additionally, some centers conducted only preoperative virus tests (14/20), while others performed both preoperative and postoperative virus tests (6/20), and none of the centers solely performed postoperative testing.

### 3.3 Treatment

In the first part of the questionnaire survey, which focused on treatment, 16 out of 20 centers reported administering AVT. Three centers relied solely on CMV-IgM test results as an indicator of AVT, while 2 centers used CMV-DNA test results alone. The majority of centers (10/16) utilized both the antibody and DNA test results as indicators of AVT. Ganciclovir was the most commonly used antiviral drug with dosages of 5 mg/kg/d (5/16) and 10 mg/kg/d (6/16), and the treatment duration was commonly 1–2 weeks (11/16). Treatment was primarily administered before the operation. And most centers (9/16) discontinued AVT once the treatment duration had ended (Figure 1C, Supplementary Table S2). Moreover, most centers (13/20) also implemented glucocorticoid therapy, with an initial dosage of 4 mg/kg/d.

### 3.4 Prognosis

In the second part of the survey, the prognostic indicators primarily include the JC rate and NLS rate, which are directly reported as percentages (%) in the survey. A total of 16 out of 20 centers surveyed illustrated the rate of JC in infants with BA after KPE. Among these, 8 centers (8/16) reported a positive group with JC rate of ≥60%. However, 13 centers (13/16) reported a JC rate greater than 60% in infants with BA with negative group (Supplementary Table S3). The rate of one-year NLS was calculated in 17 centers. Among these, 70.6% of the centers (12/17) reported a NLS rate of ≥70% in the negative group, while 52.9% of the centers (9/17) reported this rate in the positive group (Supplementary Table S3).

### 3.5 Outcomes of different groups

**Figure 2:**
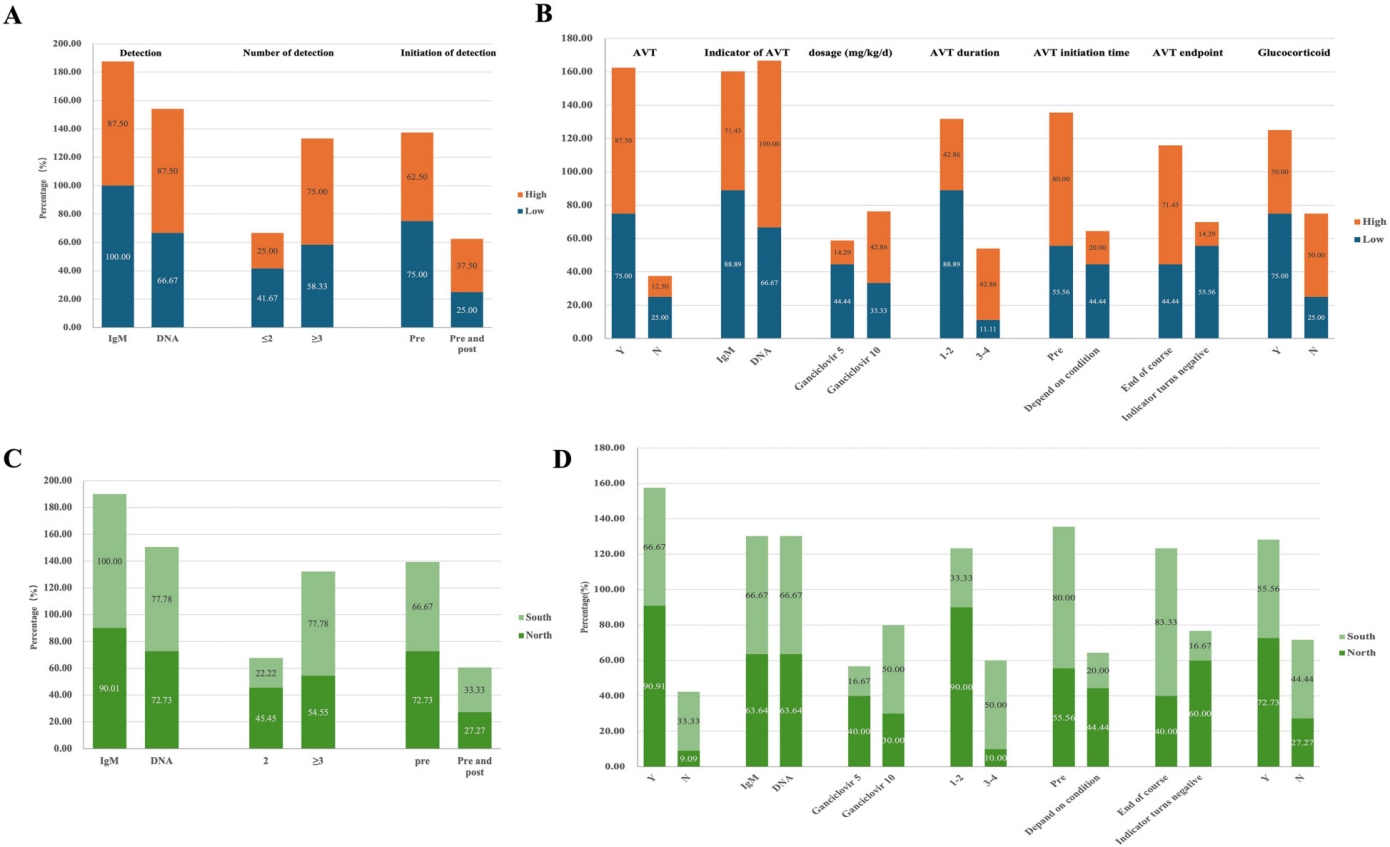
Diagnosis and treatment of CMV infected infants with BA in different subgroups. **(A)** CMV detection in low-volume vs. high-volume centers. The proportion of centers selecting different CMV detection methods among those participating in the survey, categorized by patient volume (High vs. Low). **(B)** Treatment of infants with CMV^+^ BA in low-volume vs. high-volume centers. The proportion of centers selecting various treatment approaches among those participating in the survey, categorized by patient volume (High vs. Low). **(C)** CMV detection of infants with BA in South vs. North groups. The proportion of centers selecting different CMV detection methods among those participating in the survey, categorized by geographic region (Sou vs. Nor). **(D)** Treatment of infants with CMV^+^ BA in South vs. North groups. The proportion of centers selecting different treatment approaches among those participating in the survey, categorized by geographic region (Sou vs. Nor).

The survey revealed that 75% of centers in the high-volume group (6/8) utilized three or more methods for CMV detection, compared to 58.3% (7/12) in the low-volume group. CMV-DNA detection results served as the AVT indicator in most high-volume centers (High: 100.0% vs. Low: 66.7%). In contrast, the low-volume group had a higher reliance on the CMV-IgM test for detecting viral infection (High: 87.5% vs*.* Low 100.0%) and used it as the main indicator for AVT (High: 71.4% vs. Low: 88.9%) (Figures 2A,B). Eight low-volume centers preferred a treatment duration of 1–2 weeks of AVT vs. only one center opting for 3–4 weeks, while high-volume centers showed no predominant preference for treatment duration (1–2 w: 42.9% vs. 3–4 w: 42.9%). Moreover, more centers in the high-volume group chose a dosage of 10 mg/kg/d for ganciclovir treatment, while more centers in the low-volume group opted a dosage of 5 mg/kg/d. Additionally, more medical centers in the low-volume group administered glucocorticoid therapy to infants with BA and CMV infection (High: 50.0% vs. Low: 75.0%) (Figure 2B, Supplementary Table S4).

According to the regional grouping investigation, the south group exhibited a more diverse range of detection methods for CMV in infants with BA (Sou: 77.8% vs. Nor: 54.6%) (Figures 2C,D). More centers in north group utilized AVT (Sou: 66.7% vs. Nor: 90.9%) and preferred low-dose short-cycle treatment (Dose, Sou: 16.7% vs. Nor: 40.0%; Cycle, Sou: 33.3% vs. Nor: 90.0%). More centers in south group that chose to administer treatment before surgery (Sou: 80.0% vs. Nor: 55.6%) and to discontinue AVT after the completion of therapy (Sou: 83.3% vs. Nor: 40.0%) (Figure 2D, Supplementary Table S5).

### 3.6 Retrospective analysis of 100 pediatric cases

A retrospective analysis of 100 randomly selected pediatric BA cases revealed that 39 were CMV-positive, with 29 of these infants receiving AVT. Among the CMV-positive cases, 29 tested positive for CMV-IgM, 25 for CMV-DNA, and 17 were dual-positive for both CMV-IgM and CMV-DNA. The analysis indicated that the age of Kasai was significantly higher in infants with CMV-positive BA compared to CMV-negative infants [68 days [59–76] vs. 57 days [45–69.5], *p* = 0.005] (Table 1). No significant difference in prognosis was observed between infants with CMV-positive and CMV-negative BA, likely due to the AVT received by most CMV-positive infants. Further comparison of the effects of AVT effect revealed a significant reduction in the incidence of early cholangitis in infants who received AVT (10.3% vs. 50.0%, *p* = 0.016) (Table 2).

**Table 1:** Comparison of preoperative blood examination.

Variables	Infants with BA		
	CMV^−^ BA	CMV^+^ BA	*p*-value
Age of Kasai (d)	57 (45–69.5)	68 (59–76)	0.005*
HB (g/L)	103 (96–118.5)	102 (98–110)	0.562
WBC (*10^9^/L)	10.51 (8.225–12.605)	11.285 (7.98–14.47)	0.392
PLT (*10^9/^L)	385 (267.5–506.5)	434 (333.5–514)	0.189
ALT (U/L)	109 (71–183.5)	154 (98–213)	0.056
AST (U/L)	184 (127.5–279)	240 (195–324)	0.03*
GGT (U/L)	399 (258–785.5)	429 (235–935)	0.493
ALP (U/L)	532 (434.5–719.5)	659 (434–830)	0.159
TB (umol/L)	177.5 (147.95–233.75)	196.2 (175.8–227.6)	0.05
DB (umol/L)	113.4 (101.05–142.2)	128 (109–152.7)	0.065
TBA (umol/L)	109.3 (88–133.2)	115.3(92.6–137)	0.478

HB, hemoglobin; WBC, white blood cell; PLT, platelet; ALT, alanine aminotransferase; AST, aspartate aminotransferase; GGT, gamma-glutamyl transferase; ALP, alkaline phosphatase; TB, total bilirubin; DB, direct dilirubin; TBA, total bile acids; CMV, cytomegalovirus; BA, biliary atresia.

*p-values < 0.05 were considered statistically significant.

**Table 2:** Comparison of prognosis between AVT group and non-AVT group.

Prognosis	Infants with CMV associated BA		
	AVT	non-AVT	*p* value
Jaundice Clearance			
Yes	17	5	0.721
No	12	5	
Early Cholangitis			
Yes	3	5	0.016*
No	26	5	
Frequent Cholangitis			
Yes	6	2	0.99
No	23	8	
1y-NLS			
Yes	20	8	0.693
No	9	2	

NLS, native liver survival; AVT, antiviral treatment.

*p-values < 0.05 were considered statistically significant.

## 4 Discussion

CMV is considered to be one of the possible causes of BA, and its effects on infants with BA are consistently discussed. CMV infection may influence the prognosis of BA, with some reports suggesting that AVT can improve it (14). However, there are differences in the diagnosis and treatment approaches for infants with CMV-infected BA. Through this investigation, it was found that the rate of infants with CMV-infected BA in China was approximately 31.3%. While all centers detected CMV in infants with BA, not all centers administered AVT. The retrospective analysis of 100 randomly selected BA patients indicated that AVT was beneficial for the prognosis of CMV-positive patients. There were variations in virus detection and AVT among different centers.

### 4.1 Is there a difference between centers in China?

This study surveyed 20 centers, representing half of the provinces in China. However, some provinces lack the capacity to perform Kasai, leading to the patient referrals to neighboring provinces. Therefore, the 20 centers surveyed are considered highly representative. China is a vast country with significant economic and cultural differences across regions, which can influence clinical testing methods and treatment choices. High-volume groups tended to employ multiple methods for detecting CMV infection primarily relying on CMV-DNA detection, which was used as the main indicator for AVT. In terms of AVT, most medical centers in the high-volume groups preferred high-dose ganciclovir, while those in the low-volume groups tended to choose low-dose. When comparing centers based on their geographical location (north vs. south), differences were also observed in the survey results. Centers with a higher patient volume were more likely to adopt CMV-DNA testing, as this method was more expensive and technically demanding compared to antibody testing. Notably, CMV infection was identified in approximately 30% of BA across both geographic and volume stratifications. This represented a significant decrease from previous reports (∼60%) (11) and aligns more closely with findings from countries such as the United Kingdom and Sweden, where rates range from 10% to 38% (5, 9).

### 4.2 Which indicator is more appropriate for CMV detection?

The gold standard for diagnosing CMV is virus isolation, however, this method is challenging to perform. Therefore, various diagnostic methods are used, including CMV-DNA, antibodies, or antigens testing. A positive CMV-IgM result indicates a recent infection, while CMV-DNA serves as an early marker of active infection. CMV-DNA detection relied on PCR technology to amplify the virus, offering higher sensitivity compared to CMV antibody testing (17). CMV-DNA can be detected in blood, plasma, urine, or saliva and is widely utilized (18). In Ross's study (19), it was found that the sensitivity of CMV-DNA detection in urine samples was 96.3%, with a specificity of 100%. Other reports on the sensitivity of CMV-DNA detection also show a range between 93% and 100% (20, 21). These CMV detection methods were primarily conducted using blood or urine samples, which may not fully reflect the actual status of viral infection within the liver (22). Reports of CMV inclusion bodies in the livers of infants with BA were extremely rare (23–25). In the study by Domiati-Saad (23), CMV DNA was detected by PCR in liver tissues from BA; however, no inclusion bodies were observed by hematoxylin and eosin (H&E) staining. In Xu's study (11), among 85 infants with BA, 51 tested positive for CMV DNA in liver tissue, and CMV-pp65 staining was also positive. Fischler (26) performed immunohistochemical staining for CMV-IgM and CMV-IgG in BA liver tissue and found demonstrable staining in both canalicular and sinusoidal regions. These findings suggest that viral proteins may be more readily detectable in the liver of infants with BA. Although the presence of CMV in body fluids didn't completely represent hepatic infection, detection in blood or urine was more feasible, non-invasive, and convenient for therapeutic monitoring, making it more acceptable in clinical practice.

Antibody tests, such as CMV-IgM and CMV-IgG, are more accessible and cost-effective than other methods. However, the stability of blood sample for these tests was limited (27). The CMV-IgM test has a high false-negative rate, while CMV-IgG is less reliable for detection since it can cross the placental barrier and may originate maternal antibodies. CMV-DNA is highly sensitive, whereas CMV-pp65 can serve as an indicator of active CMV infection (28). Although CMV-DNA is a key marker for CMV detection, its high technical requirements and associated cost have led many centers to prefer CMV-IgM antibody testing.

Different methods of CMV detections may also indicate different prognosis for infants with CMV-infected BA. A study reported infants with CMV-pp65-positive BA had a worse prognosis than infants in the CMV antibody-positive group (13). However, current evidence suggest that the use of CMV-pp65 testing remained far less common than CMV-DNA or CMV antibody testing, likely due to its higher technical demands and associated costs.

### 4.3 Is antiviral therapy necessary?

AVT is commonly administered before surgery, which undoubtedly delays the age of Kasai. Previous studies have also reported that infants with CMV-positive BA tend to undergo the Kasai at an older age (6, 29). The age of Kasai was an important factor influencing the prognosis of infants with BA (30). This raises the question: should AVT be routinely administered to infants with CMV-infected BA?.

According to the European consensus, infants with congenital CMV infection who exhibit central nervous system damage, severe single-system damage, or multisystem damage may require regular AVT (18). Congenital CMV-infected infants are at risk of sensorineural hearing loss (SNHL) during childhood, whereas congenital CMV infection was defined as CMV infection determined within the first 2–3 weeks of birth. The consensus suggest that CMV-infected infants undergo systematic evaluation to determine the necessity of treatment, and most infants do not require AVT.

Some pediatric surgeons argued that infants with BA were distinct from normal infants and should be considered separately when deciding on AVT. Since most infants with BA were tested in the hospital at an age older than 14 days, it was difficult to determine whether the infection was congenital. The hepatic immune environment differs between infants with BA and healthy infants, and CMV infection also affects immune cells in BA (31). CMV infection also affect the prognosis of infants with BA (9, 14). AVT had been shown to improve the prognosis of CMV-infected infants with BA. A European multicenter study demonstrated that the NLS in infants with CMV-infected BA who received AVT was significantly better than that in CMV-infected BA who did not receive AVT (14). In another study, antiviral drugs were found to attenuate the pathogenic effects of CMV, improved JC, and reduced the incidence of cholangitis. Given these findings, some experts in in-person conference communication believed that postoperative AVT could be considered for patients who were in poor general condition and older in age.

### 4.4 How can antiviral treatment be more effective?

There are currently no uniform guidelines for the treatment of CMV-infected BA patients. Ganciclovir and valganciclovir were the main drugs used for treating AVT (32). In a Swedish retrospective study, 46.13% (6/13) of infants were treated with valganciclovir, while 30.8% (4/13) of infants received ganciclovir, and the remaining infants were treated sequentially. The dosage of ganciclovir varied between 5 and 11 mg/kg/d, with the duration ranging from 21 to 74 days (14). In a UK study, all CMV-infected infants with BA received preoperative AVT. Among these, some (5/8) received ganciclovir (5 mg/kg/d), and some infants received oral valganciclovir (520 mg/m^2^). The use of valganciclovir treatment was more common in Western countries, whereas ganciclovir usage was similar in Eastern and Western countries. Ganciclovir was a nucleotide analog antiviral drug that inhibited viral replication, but it was associated with certain side effects, including neurotoxicity, bone marrow suppression, liver damage, and allergic reactions (33, 34). Further investigation is needed to determine whether ganciclovir treatment may exacerbate liver damage.

### 4.5 Do CMV-infected infants with BA need special attention at the time of review?

The latent-activation characteristics of CMV and changes in the immune microenvironment were common in infants with BA (31). There was also a possibility of viral activation during the follow-up. Therefore, the author believed that regular postoperative reviews of CMV-infected infants with BA were necessary. The treatment of CMV relapse in CMV-infected BA has been discussed, but there are fewer references available, and further investigations are needed.

### 4.6 Limitation

This study had several limitations. Firstly, there may be differences among participating centers in the way of diagnosing, treating, and following up. Secondly, this study relied on questionnaires, which were prone to recall bias. Thirdly, data were collected from 20 medical centers, which may not fully represent the diagnostic and treatment practices for CMV-infected infants with BA across mainland China. Further studies involving a broader range of hospitals were needed to achieve more representative results. Finally, in the second part of the questionnaire, each center reported prognostic outcomes rather than providing detailed clinical data for each patient, resulting in a lack of comprehensive clinical information.

## 5 Conclusion

In conclusion, this study highlighted that most centers in China recognized the significance of CMV infection in infants with BA. However, there were currently no uniform standards for detection and AVT. This indicated a need for multicenter retrospective and prospective studies. The descriptive study conducted through the questionnaire investigation provided a foundation for further in-depth research in this area.

## Data Availability

The original contributions presented in the study are included in the article/Supplementary Material, further inquiries can be directed to the corresponding author.

## References

[1] Bezerra JA Wells RG Mack CL Karpen SJ Hoofnagle JH Doo E Biliary atresia: clinical and research challenges for the twenty-first century. Hepatology. (2018) 68(3):1163–73. 10.1002/hep.29905 29604222 PMC6167205

[2] Asai A Miethke A Bezerra JA. Pathogenesis of biliary atresia: defining biology to understand clinical phenotypes. Nat Rev Gastroenterol Hepatol. (2015) 12(6):342–52. 10.1038/nrgastro.2015.74 26008129 PMC4877133

[3] Davenport M. Biliary atresia: clinical aspects. Semin Pediatr Surg. (2012) 21(3):175–84. 10.1053/j.sempedsurg.2012.05.010 22800970

[4] Chiu CY Chen PH Chan CF Chang MH Wu TC. Biliary atresia in preterm infants in Taiwan: a nationwide survey. J Pediatr. (2013) 163(1):100–3.e1. 10.1016/j.jpeds.2012.12.085 23414661

[5] Lakshminarayanan B Davenport M. Biliary atresia: a comprehensive review. J Autoimmun. (2016) 73:1–9. 10.1016/j.jaut.2016.06.005 27346637

[6] Murase N Hinoki A Shirota C Tomita H Shimojima N Sasaki H Multicenter, retrospective, comparative study of laparoscopic and open Kasai portoenterostomy in children with biliary atresia from Japanese high-volume centers. J Hepatobiliary Pancreat Sci. (2019) 26(1):43–50. 10.1002/jhbp.594 30488647

[7] Ge L Zhan J Gao W Zhao S Xu X Dou R. Relevant factors for early liver transplantation after Kasai portoenterostomy. BMC Pediatr. (2020) 20(1):484. 10.1186/s12887-020-02355-8 33081738 PMC7574207

[8] Ortiz-Perez A Donnelly B Temple H Tiao G Bansal R Mohanty SK. Innate immunity and pathogenesis of biliary atresia. Front Immunol. (2020) 11:329. 10.3389/fimmu.2020.00329 32161597 PMC7052372

[9] Zani A Quaglia A Hadzić N Zuckerman M Davenport M. Cytomegalovirus-Associated biliary atresia: an aetiological and prognostic subgroup. J Pediatr Surg. (2015) 50(10):1739–45. 10.1016/j.jpedsurg.2015.03.001 25824438

[10] Soomro GB Abbas Z Hassan M Luck N Memon Y Khan AW. Is there any association of extra hepatic biliary atresia with cytomegalovirus or other infections? J Pak Med Assoc. (2011) 61(3):281–3. 21465946

[11] Xu Y Yu J Zhang R Yin Y Ye J Tan L The perinatal infection of cytomegalovirus is an important etiology for biliary atresia in China. Clin Pediatr (Phila). (2012) 51(2):109–13. 10.1177/0009922811406264 22144720

[12] Liliemark U Svensson JF Fischler B. Incidence and antiviral treatment of cytomegalovirus infection in infants with biliary atresia. Pediatr Surg Int. (2023) 39(1):117. 10.1007/s00383-023-05394-1 36773050

[13] Shen C Zheng S Wang W Xiao XM. Relationship between prognosis of biliary atresia and infection of cytomegalovirus. World J Pediatr. (2008) 4(2):123–6. 10.1007/s12519-008-0024-8 18661768

[14] Fischler B Czubkowski P Dezsofi A Liliemark U Socha P Sokol RJ Incidence, impact and treatment of ongoing cmv infection in patients with biliary atresia in four European centres. J Clin Med. (2022) 11(4):945. 10.3390/jcm11040945 35207217 PMC8879500

[15] Zhao Y Xu X Liu G Yang F Zhan J. Prognosis of biliary atresia associated with cytomegalovirus: a meta-analysis. Front Pediatr. (2021) 9:710450. 10.3389/fped.2021.710450 34490166 PMC8416545

[16] Parolini F Hadzic N Davenport M. Adjuvant therapy of cytomegalovirus igm + Ve associated biliary atresia: prima facie evidence of effect. J Pediatr Surg. (2019) 54(9):1941–5. 10.1016/j.jpedsurg.2018.12.014 30772005

[17] Özkarata E Özbek ÖA Avkan Oğuz V Sayıner AA. Comparison of the cmv antigenemia test and cmv-DNA pcr results in solid organ transplant recipients. Mikrobiyol Bul. (2016) 50(1):44–52. 10.5578/mb.10701 27058328

[18] Luck SE Wieringa JW Blázquez-Gamero D Henneke P Schuster K Butler K Congenital cytomegalovirus: a European expert consensus statement on diagnosis and management. Pediatr Infect Dis J. (2017) 36(12):1205–13. 10.1097/inf.0000000000001763 29140947

[19] Ross SA Ahmed A Palmer AL Michaels MG Sánchez PJ Bernstein DI Detection of congenital cytomegalovirus infection by real-time polymerase chain reaction analysis of Saliva or urine specimens. J Infect Dis. (2014) 210(9):1415–8. 10.1093/infdis/jiu263 24799600 PMC4271051

[20] Tsai CH Tsai FJ Shih YT Wu SF Liu SC Tseng YH. Detection of congenital cytomegalovirus infection in Chinese newborn infants using polymerase chain reaction. Acta Paediatr. (1996) 85(10):1241–3. 10.1111/j.1651-2227.1996.tb18237.x 8922092

[21] de Vries JJ van der Eijk AA Wolthers KC Rusman LG Pas SD Molenkamp R Real-Time pcr versus viral culture on urine as a gold standard in the diagnosis of congenital cytomegalovirus infection. J Clin Virol. (2012) 53(2):167–70. 10.1016/j.jcv.2011.11.006 22177273

[22] Fischler B Ehrnst A Forsgren M Orvell C Nemeth A. The viral association of neonatal cholestasis in Sweden: a possible link between cytomegalovirus infection and extrahepatic biliary atresia. J Pediatr Gastroenterol Nutr. (1998) 27(1):57–64. 10.1097/00005176-199807000-00010 9669727

[23] Domiati-Saad R Dawson DB Margraf LR Finegold MJ Weinberg AG Rogers BB. Cytomegalovirus and human herpesvirus 6, but not human papillomavirus, are present in neonatal giant cell hepatitis and extrahepatic biliary atresia. Pediatr Dev Pathol. (2000) 3(4):367–73. 10.1007/s100240010045 10890252

[24] Tarr PI Haas JE Christie DL. Biliary atresia, cytomegalovirus, and age at referral. Pediatrics. (1996) 97(6 Pt 1):828–31. 10.1542/peds.97.6.828 8657522

[25] Chan ES Abou Mehrem A de Koning L Stritzke A Zhou HY. Extrahepatic biliary atresia in a premature neonate with congenital cytomegalovirus infection. Pathology. (2023) 55(4):573–6. 10.1016/j.pathol.2022.10.013 36774239

[26] Fischler B Woxenius S Nemeth A Papadogiannakis N. Immunoglobulin deposits in liver tissue from infants with biliary atresia and the correlation to cytomegalovirus infection. J Pediatr Surg. (2005) 40(3):541–6. 10.1016/j.jpedsurg.2004.11.035 15793732

[27] Kamei H Ito Y Onishi Y Suzuki M Imai H Kurata N Cytomegalovirus (CMV) monitoring after liver transplantation: comparison of CMV Pp65 antigenemia assay with real-time PCR calibrated to who international standard. Ann Transplant. (2016) 21:131–6. 10.12659/aot.895677 26927444

[28] Caliendo AM St George K Kao SY Allega J Tan BH LaFontaine R Comparison of quantitative cytomegalovirus (cmv) pcr in plasma and cmv antigenemia assay: clinical utility of the prototype amplicor cmv monitor test in transplant recipients. J Clin Microbiol. (2000) 38(6):2122–7. 10.1128/jcm.38.6.2122-2127.2000 10834964 PMC86743

[29] Fischler B Svensson JF Nemeth A. Early cytomegalovirus infection and the long-term outcome of biliary atresia. Acta Paediatr. (2009) 98(10):1600–2. 10.1111/j.1651-2227.2009.01416.x 19604167

[30] Nio M Sasaki H Wada M Kazama T Nishi K Tanaka H. Impact of age at Kasai operation on short- and long-term outcomes of type III biliary atresia at a single institution. J Pediatr Surg. (2010) 45(12):2361–3. 10.1016/j.jpedsurg.2010.08.032 21129545

[31] Hill R Quaglia A Hussain M Hadzic N Mieli-Vergani G Vergani D Th-17 cells infiltrate the liver in human biliary atresia and are related to surgical outcome. J Pediatr Surg. (2015) 50(8):1297–303. 10.1016/j.jpedsurg.2015.02.005 25783388

[32] Fischler B Casswall TH Malmborg P Nemeth A. Ganciclovir treatment in infants with cytomegalovirus infection and cholestasis. J Pediatr Gastroenterol Nutr. (2002) 34(2):154–7. 10.1097/00005176-200202000-00009 11840032

[33] Mareri A Lasorella S Iapadre G Maresca M Tambucci R Nigro G. Anti-viral therapy for congenital cytomegalovirus infection: pharmacokinetics, efficacy and side effects. J Matern Fetal Neonatal Med. (2016) 29(10):1657–64. 10.3109/14767058.2015.1058774 26135794

[34] Chiopris G Veronese P Cusenza F Procaccianti M Perrone S Daccò V Congenital cytomegalovirus infection: update on diagnosis and treatment. Microorganisms. (2020) 8(10):1516. 10.3390/microorganisms8101516 33019752 PMC7599523

