# Supplementary material for: Cytomegalovirus infection in infants with biliary atresia in China: a multi-center investigation study
Source: Front Pediatr. 2025 Jun 6;13:1577113. doi: 10.3389/fped.2025.1577113 (PMC12179059; doi:10.3389/fped.2025.1577113)
Supplement: Supplementary file 1 [file Datasheet1.docx]

Investigation of cytomegalovirus infection

in biliary atresia patients

Dear experts and professors, in order to better understand the infection, diagnosis and treatment of cytomegalovirus (CMV) in biliary atresia (BA) patients in mainland China. We intend to collect data on cytomegalovirus infected biliary atresia patients in each center for two years from 2018.1.1 to 2020.1.1. Thank you for taking time out of your busy schedules to fill out this questionnaire.

Part I: Basic information

1. Organization

2. Department

3.Name

4. Related tests for CMV (multiple choice) ( )

A. CMV-IgM B. CMV-IgG C. CMV-DNA (blood or urine)

D. CMV-pp65 E. CMV Inclusion Body F. Other (Fill in)

5. Time to perform virus detection ( )

A. Pre-operative only B. Post-operative only C. Pre-operative and post-operative

6. Number of BA patients admitted from 1.1.2018 to 1.1.2020

7. Number of CMV positive children admitted from 1.1.2018 to 1.1.2020

8. Criteria for BA patients on antiviral therapy (AVT) (multiple choice) ( )

A. Positive CMV-IgM B. Positive CMV-pp65 test C. Positive CMV-DNA (blood or urine) test D. 1 positive test for CMV-IgG E. 2 positive tests for CMV-IgG

F. Other ( )

9. Whether to administer AVT to CMV infected BA patients ( )

A. Yes B. No (Continue if yes, skip to 17 if no)

10. AVT commonly used drugs for (multiple choice)( )

A. ganciclovir (dose: mg/kg/d) B. valganciclovir (dose: mg/kg/d)

C. ganciclovir and valganciclovir in sequence (dose: mg/kg/d)

D. Chinese medicine E. Other drugs ( )

11. AVT course ( )

A. 1-2 weeks B. 3-4 weeks C. 1-2 months

D. 3-4 months E. 5-6 months D. Other ( )

12. Is preoperative viral testing performed ( )

A. Yes B. No

13. If a child is tested CMV positive preoperatively, is AVT administered preoperatively ( )

A. Yes B. No

C. Depending on the child, some cases of surgery followed by antiviral therapy

14. Whether to perform postoperative viral testing ()

A. Yes B. No

15. If the postoperative test is positive, is postoperative AVT administered ( )

A. Yes B. No

16. Time of discontinuation of antiviral therapy ()

A. the end of the course of treatment B. detection of CMV-DNA to turn negative

C. detection of CMV-pp65 to turn negative D. CMV-IgM test is negative

17. Will the CMV infected BA infants be treated with hormonal adjuvant therapy ( )

A. Yes (If yes, continue to answer ) B. No (If no, skip to question 19)

18. The starting dose of hormonal adjuvant therapy in CMV infected BA infants is ( )

A. 10 mg/Kg/d B. 4 mg/Kg/d C. 2 mg/Kg/d

D. 1 mg/Kg/d E. Other ( )

19. The average age of surgery in infants with CMV infected BA is ( )

A. ≤50d B. 51-60d C. 61-70d D. 71-80d E. 81-90d F. >90d

20. The average age of surgery in infants with CMV non-infected BA is

A. ≤50d B. 51-60d C. 61-70d D. 71-80d E. 81-90d F. >90d

21. Whether to continue CMV tests during the postoperative review in infants with CMV infected BA ( ) (choice A or C please answer question 22)

A. Yes B. No C. Not necessarily (describe the situation)

22. Which of the following viral tests is mostly used when reviewing a CMV infected BA infants ( ) (multiple choice)

A. CMV-IgM B. CMV-IgG C. CMV-DNA (blood or urine)

D. CMV-pp65 E. CMV inclusion bodies F. Other ( )

23. If retest is positive for CMV, is antiviral therapy administered? ( )

A. Yes, continue treatment according to preoperative treatment

B. Yes, take other doses (instructions) C. No, no more antiviral treatment

**Part II Prognosis**

Define CMV-positive BA infants. That is, BA infants were defined as a CMV-positive BA if any of the following indicators are met: (1) positive blood or urine CMV-DNA; (2) positive blood or urine CMV-pp65; (3) positive blood CMV-IgM; (4) ≥4-fold increase in antibody titers to double serum CMV-IgG; (5) positive urine cytomegalovirus inclusion bodies.

1. The rate of jaundice clearance in CMV positive BA infants is ( )%

Note: Jaundice clearance is defined as a decrease in total bilirubin to less than 20 umol/L within 6 months.
2. The rate of jaundice clearance in CMV negative BA infants is ( )%

3. The incidence of early cholangitis in infants with CMV positive BA is ( )%

Note: Cholangitis occurring within 1 month after surgery is considered early cholangitis

4. The incidence of early cholangitis in infants with CMV negative BA is ( )%

5. The incidence of frequent cholangitis in infants with CMV positive BA is ( )%

Note: Frequent cholangitis is defined as ≥3 episodes of cholangitis within 6 months after surgery

6. The incidence of frequent cholangitis in infants with CMV negative BA was ( )%

7. 1-year native liver survival in infants with CMV positive BA ( )%

8. 1-year native liver survival in infants with CMV negative BA ( )%
