# Supplementary figures and images for: Cytomegalovirus infection in infants with biliary atresia in China: a multi-center investigation study
Source: Front Pediatr. 2025 Jun 6;13:1577113. doi: 10.3389/fped.2025.1577113 (PMC12179059; doi:10.3389/fped.2025.1577113)

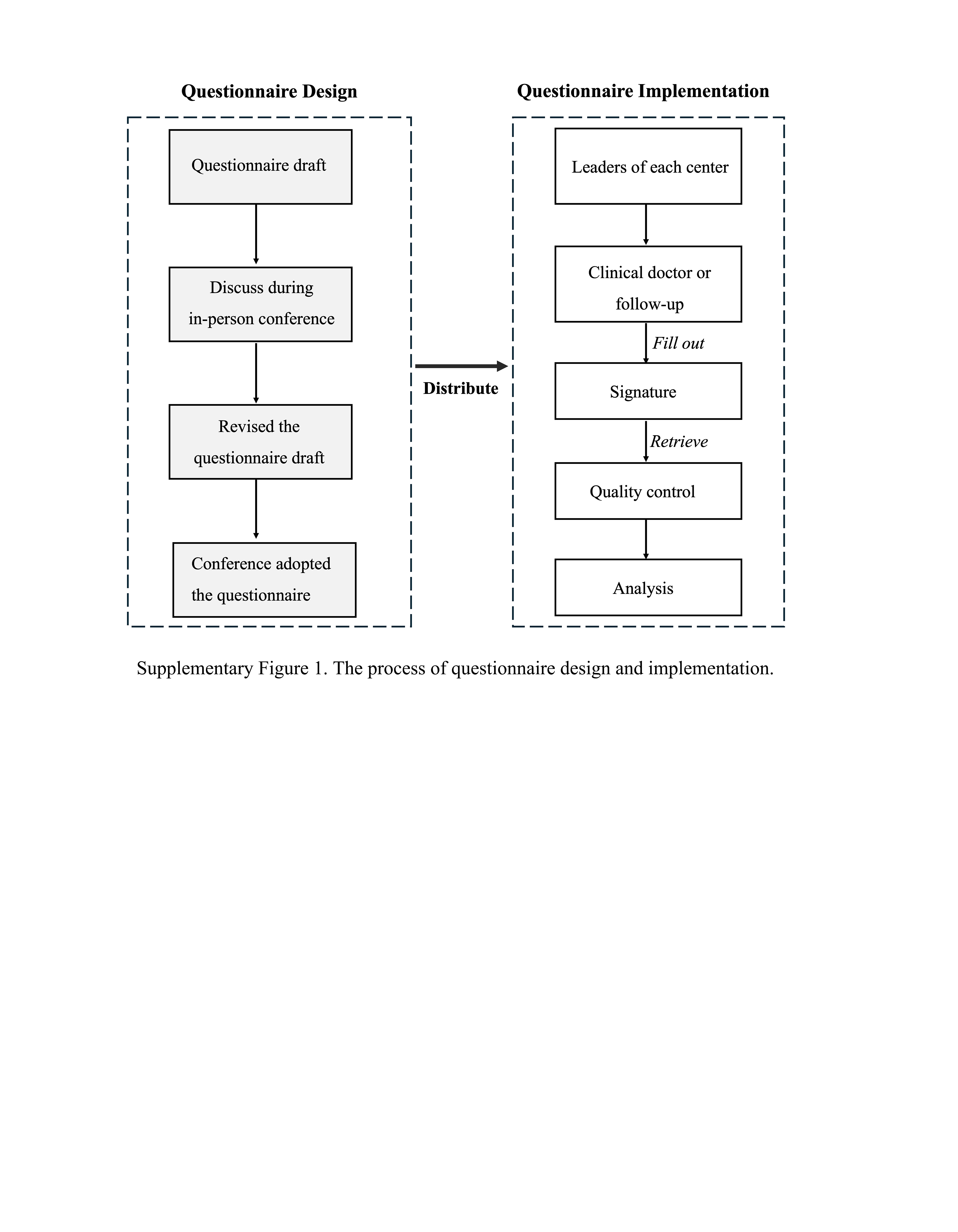

Supplement: Supplementary file 2 [file Image1.tif]
