# Supplementary material for: Cytomegalovirus infection in infants with biliary atresia in China: a multi-center investigation study
Source: Front Pediatr. 2025 Jun 6;13:1577113. doi: 10.3389/fped.2025.1577113 (PMC12179059; doi:10.3389/fped.2025.1577113)
Supplement: Supplementary file 3 [file Table1.docx]

S1 Table. Involved Center Information

| Num | Geography | Patient caseload | CMV detection | Detection time | Num of BA  (n) | Num of CMV infected BA  [n (%)] | Age of Kasai in positive Group | Age of Kasai in negative Group |
| --- | --- | --- | --- | --- | --- | --- | --- | --- |
| 1 | Nor | low | CMV-IgM\CMV-IgG\CMV-DNA(blood/urine) | Pre | 50 | 18 (36%) | 71-80d | 61-70d |
| 2 | Sou | low | CMV-IgM/CMV-IgG/CMV-pp65/CMV-inclusion body | Pre | 20 | 12 (60%) | 51-60d | ≤50d |
| 3 | Sou | high | CMV-IgM/CMV-IgG/CMV-DNA(blood/urine) | Pre and Post | 60 | 20 (33.33%) | 51-60d | ≤50d |
| 4 | Nor | low | CMV-IgM/CMV-IgG | Pre | 50 | 0 (0%) | - | - |
| 5 | Nor | low | CMV-IgM/CMV-IgG/CMV-DNA(blood/urine)/CMV-pp65 | Pre and Post | 28 | 3 (10.71%) | 51-60d | 51-60d |
| 6 | Nor | low | CMV-IgM/CMV-IgG/CMV-DNA(blood/urine) | Pre | 8 | 5 (62.5%) | ≤50d | ≤50d |
| 7 | Sou | low | CMV-IgM/CMV-IgG/CMV-DNA(blood/urine) | Pre and Post | 20 | 6 (30%) | 61-70d | 51-60d |
| 8 | Sou | high | CMV-IgM/CMV-IgG/CMV-DNA(blood/urine) | Pre | 68 | 32 (47.06%) | 61-70d | 51-60d |
| 9 | Nor | low | CMV-IgM/CMV-DNA(blood/urine) | Pre and Post | 40 | 15 (37.5%) | 61-70d | 51-60d |
| 10 | Sou | high | CMV-IgM/CMV-IgG/CMV-DNA(blood/urine)/Others [CMV-DNA (breast milk)] | Pre | 52 | 7 (13.46%) | 61-70d | 51-60d |
| 11 | Sou | low | CMV-IgM/CMV-DNA(blood/urine) | Pre | 47 | 12 (25.53%) | 71-80d | 61-70d |
| 12 | Sou | high | CMV-IgM/CMV-IgG/CMV-DNA(blood/urine)/CMV-inclusion body | Pre and Post | 300 | 100 (33.33%) | ≤50d | ≤50d |
| 13 | Nor | high | CMV-DNA(blood/urine) | Pre and Post | 200 | 50 (25%) | ≤50d | ≤50d |
| 14 | Sou | high | CMV-IgM/CMV-IgG/CMV-DNA(blood/urine) | Pre | 56 | 18 (32.14%) | 61-70d | 51-60d |
| 15 | Nor | low | CMV-IgM/CMV-IgG/CMV-DNA (blood/urine) | Pre | 26 | 7 (26.92%) | 61-70d | 71-80d |
| 16 | Nor | low | CMV-IgM/CMV-IgG/CMV-DNA(blood/urine)/CMV-pp65/CMV-inclusion body | Pre | 22 | 9 (40.91%) | 61-70d | 61-70d |
| 17 | Nor | low | CMV-IgM/CMV-IgG | Pre | 8 | 2 (25%) | 71-80d | 61-70d |
| 18 | Sou | high | CMV-IgM | Pre | 72 | 15 (20.83%) | ≤50d | ≤50d |
| 19 | Nor | low | CMV-IgM | Pre | 45 | 16 (35.56%) | 51-60d | 61-70d |
| 20 | Sou | high | CMV-IgM/CMV-IgG/CMV-DNA(blood/urine) | Pre | 104 | 52 (50%) | ≤50d | 51-60d |
| 21 | - | - | CMV-IgM/CMV-IgG | Pre | 25 | 30 | ≤50d | ≤50d |
| 22 | - | - | CMV-IgM/CMV-IgG | Pre | 0 | 0 | ≤50d | ≤50d |

CMV, cytomegalovirus; BA, Biliary atresia; Pre, Preoperative; Post, Postoperative.
