# Supplementary material for: Cytomegalovirus infection in infants with biliary atresia in China: a multi-center investigation study
Source: Front Pediatr. 2025 Jun 6;13:1577113. doi: 10.3389/fped.2025.1577113 (PMC12179059; doi:10.3389/fped.2025.1577113)
Supplement: Supplementary file 4 [file Table2.docx]

S2 Table. Adjuvant therapy for CMV-infected BA in multicenter in China

| Index |  | Num | Proportion |
| --- | --- | --- | --- |
| CMV infection | CMV positive BA | 399 | 31.27% |
|  | CMV negative BA | 877 | 68.73% |
|  |  |  |  |
| Indicator of AVT | IgM | 13/16 | 81.25% |
|  | DNA | 13/16 | 81.25% |
|  |  |  |  |
| AVT | Y | 16/20 | 80.00% |
|  | N | 4/20 | 20.00% |
|  |  |  |  |
| AVT dosage  (mg/kg/d) | Ganciclovir 5 | 5/16 | 31.25% |
|  | Ganciclovir 10 | 6/16 | 37.50% |
|  | Sequential treatment | 1/16 | 6.25% |
|  |  |  |  |
| AVT dosage duration (w) | 1-2 | 11/16 | 68.75% |
|  | 3-4 | 4/16 | 25.00% |
|  |  |  |  |
| AVT initiation time | Preoperative | 9/14 | 64.29% |
|  | Depend on condition | 5/14 | 35.71% |
|  |  |  |  |
| AVT endpoint criteria | End of course | 9/16 | 56.25% |
|  | CMV-DNA（-） | 3/16 | 18.75% |
|  | CMV-pp65（-） | 1/16 | 6.25% |
|  | CMV-IgM（-） | 2/16 | 12.50% |
|  |  |  |  |
| Glucocorticoid | Y | 13/20 | 65.00% |
|  | N | 7/20 | 35.00% |
|  |  |  |  |
| Glucocorticoid dosage  (mg/kg/d) | 4 | 7/13 | 53.85% |

AVT, antiviral treatment; Y, yes, indicates the implementation of this treatment (antiviral or glucocorticosteroid treatment); N, no, indicates the absence of this treatment (antiviral or glucocorticosteroid treatment); Num, number; CMV, cytomegalovirus; w, week; (-) means the indicator has turned negative.
