# Supplementary material for: Cytomegalovirus infection in infants with biliary atresia in China: a multi-center investigation study
Source: Front Pediatr. 2025 Jun 6;13:1577113. doi: 10.3389/fped.2025.1577113 (PMC12179059; doi:10.3389/fped.2025.1577113)
Supplement: Supplementary file 5 [file Table3.docx]

S3 Table. Prognosis of multi-centers

| Program |  |  | Proportion | Percentage |  | Proportion | Percentage |  | Proportion | Percentage |  | Proportion | Proportion |
| --- | --- | --- | --- | --- | --- | --- | --- | --- | --- | --- | --- | --- | --- |
| JC | pos | ≥60% | 8/16 | 50.00% | ≥80% | 4/16 | 25.00% |  |  |  |  |  |  |
|  | neg | ≥60% | 13/16 | 76.47% | ≥80% | 4/16 | 23.53% |  |  |  |  |  |  |
|  |  |  |  |  |  |  |  |  |  |  |  |  |  |
| Early cholangitis | pos | ≤40% | 12/18 | 66.67% | ≤30% | 8/18 | 44.44% | ≤20% | 6/18 | 33.33% | ≤10% | 2/18 | 11.11% |
|  | neg | ≤40% | 12/18 | 66.67% | ≤30% | 12/18 | 66.67% | ≤20% | 8/18 | 44.44% | ≤10% | 2/18 | 11.11% |
|  |  |  |  |  |  |  |  |  |  |  |  |  |  |
| Frequent Cholangitis | pos | ≤40% | 13/17 | 76.47% | ≤30% | 13/17 | 76.47% | ≤20% | 11/17 | 64.71% | ≤10% | 3/17 | 17.65% |
|  | neg | ≤40% | 14/17 | 82.35% | ≤30% | 14/17 | 82.35% | ≤20% | 13/17 | 76.47% | ≤10% | 5/17 | 29.41% |
|  |  |  |  |  |  |  |  |  |  |  |  |  |  |
| NLS | pos | ≥60% | 12/17 | 70.89% | ≥70% | 9/17 | 52.94% | ≥80% | 6/17 | 35.29% |  |  |  |
|  | neg | ≥60% | 13/17 | 76.47% | ≥70% | 12/17 | 70.59% | ≥80% | 8/17 | 47.06% |  |  |  |

JC, Jaundice clearance; NLS, native liver survival; pos, CMV positive group; neg, CMV negative group; Percentage, percentage of involved centers
