# Supplementary material for: Cytomegalovirus infection in infants with biliary atresia in China: a multi-center investigation study
Source: Front Pediatr. 2025 Jun 6;13:1577113. doi: 10.3389/fped.2025.1577113 (PMC12179059; doi:10.3389/fped.2025.1577113)
Supplement: Supplementary file 6 [file Table4.docx]

S4 Table. Comparison of diagnosis and treatment of CMV-infected BA in different caseload centers

| Index |  | Low | Proportion | High | Proportion | *P* |
| --- | --- | --- | --- | --- | --- | --- |
| Detection of CMV | IgM | 12/12 | 100.00% | 7/8 | 87.50% | 0.728 |
|  | DNA | 8/12 | 66.67% | 7/8 | 87.50% |  |
|  |  |  |  |  |  |  |
| Number of detection (n) | ≤2 | 4/12 | 41.67% | 2/8 | 25.00% | 0.642 |
|  | ≥3 | 7/12 | 58.33% | 6/8 | 75.00% |  |
|  |  |  |  |  |  |  |
| Initiation time of detection | Pre | 9/12 | 75.00% | 5/8 | 62.50% | 0.642 |
|  | Pre and post | 3/12 | 25.00% | 3/8 | 37.50% |  |
|  |  |  |  |  |  |  |
| CMV infection | Y | 105 | 28.85% | 294 | 32.24% | 0.238 |
|  | N | 259 | 71.15% | 618 | 67.76% |  |
|  |  |  |  |  |  |  |
| AVT | Y | 9/12 | 75.00% | 7/8 | 87.50% | 0.619 |
|  | N | 3/12 | 25.00% | 1/8 | 12.50% |  |
|  |  |  |  |  |  |  |
| Indicator of AVT | IgM | 8/9 | 88.89% | 5/7 | 71.43% | 0.695 |
|  | DNA | 6/9 | 66.67% | 7/7 | 100.00% |  |
|  |  |  |  |  |  |  |
| AVT dosage  (mg/kg/d) | Ganciclovir 5 | 4/9 | 44.44% | 1/7 | 14.29% | 0.545 |
|  | Ganciclovir 10 | 3/9 | 33.33% | 3/7 | 42.86% |  |
|  |  |  |  |  |  |  |
| AVT dosage duration (w) | 1-2 | 8/9 | 88.89% | 3/7 | 42.86% | 0.235 |
|  | 3-4 | 1/9 | 11.11% | 3/7 | 42.86% |  |
|  |  |  |  |  |  |  |
| AVT initiation time | Pre | 5/9 | 55.56% | 4/5 | 80.00% | 0.580 |
|  | Depend on condition | 4/9 | 44.44% | 1/5 | 20.00% |  |
|  |  |  |  |  |  |  |
| AVT endpoint criteria | End of course | 4/9 | 44.44% | 5/7 | 71.43% | 0.287 |
|  | Indicator turns negative | 5/9 | 55.56% | 1/7 | 14.29% |  |
|  |  |  |  |  |  |  |
| Glucocorticoid | Y | 9/12 | 75.00% | 4/8 | 50.00% | 0.356 |
|  | N | 3/12 | 25.00% | 4/8 | 50.00% |  |

CMV, cytomegalovirus; Pre, Preoperative; Post, Postoperative; AVT, antiviral treatment; Y, yes, indicates the implementation of this treatment (antiviral treatment or glucocorticosteroid treatment); N, no, indicates the absence of this treatment (antiviral treatment or glucocorticosteroid treatment); w, week. Fisher exact probability method analyzed.
