# Supplementary material for: Cytomegalovirus infection in infants with biliary atresia in China: a multi-center investigation study
Source: Front Pediatr. 2025 Jun 6;13:1577113. doi: 10.3389/fped.2025.1577113 (PMC12179059; doi:10.3389/fped.2025.1577113)
Supplement: Supplementary file 7 [file Table5.docx]

S5 Table. Comparison of diagnosis and treatment of CMV-infected BA in diverse geographical centers

| Index |  | North group | Proportion | South  group | Proportion | *P* |
| --- | --- | --- | --- | --- | --- | --- |
| Detection of CMV | IgM | 10/11 | 90.91% | 9/9 | 100.00% | ＞0.999 |
|  | DNA | 8/11 | 72.73% | 7/9 | 77.78% |  |
|  |  |  |  |  |  |  |
| Number of detection (n) | 2 | 5/11 | 45.45% | 2/9 | 22.22% | 0.374 |
|  | ≥3 | 6/11 | 54.55% | 7/9 | 77.78% |  |
|  |  |  |  |  |  |  |
| Initiation time of detection | pre | 8/11 | 72.73% | 6/9 | 66.67% | ＞0.999 |
|  | Pre and post | 3/11 | 27.27% | 3/9 | 33.33% |  |
|  |  |  |  |  |  |  |
| CMV infected | CMV positive BA | 177 | 30.46% | 222 | 31.94% | 0.573 |
|  | CMV negative BA | 404 | 69.54% | 473 | 68.06% |  |
|  |  |  |  |  |  |  |
| AVT | Y | 10/11 | 90.91% | 6/9 | 66.67% | 0.285 |
|  | N | 1/11 | 9.09% | 3/9 | 33.33% |  |
|  |  |  |  |  |  |  |
| Indicator of AVT | IgM | 7/11 | 63.64% | 6/9 | 66.67% | ＞0.99 |
|  | DNA | 7/11 | 63.64% | 6/9 | 66.67% |  |
|  |  |  |  |  |  |  |
| AVT dosage  (mg/kg/d) | Ganciclovir 5 | 4/10 | 40.00% | 1/6 | 16.67% | 0.545 |
|  | Ganciclovir 10 | 3/10 | 30.00% | 3/6 | 50.00% |  |
|  |  |  |  |  |  |  |
| AVT dosage  duration (w) | 1-2 | 9/10 | 90.00% | 2/6 | 33.33% | 0.077 |
|  | 3-4 | 1/10 | 10.00% | 3/6 | 50.00% |  |
|  |  |  |  |  |  |  |
| AVT initiation time | Pre | 5/9 | 55.56% | 4/5 | 80.00% | 0.58 |
|  | Depend on condition | 4/9 | 44.44% | 1/5 | 20.00% |  |
|  |  |  |  |  |  |  |
| AVT endpoint criteria | End of course | 4/10 | 40.00% | 5/6 | 83.33% | 0.145 |
|  | Indicator turns negative | 6/10 | 60.00% | 1/6 | 16.67% |  |
|  |  |  |  |  |  |  |
| Glucocorticoid | Y | 8/11 | 72.73% | 5/9 | 55.56% | 0.642 |
|  | N | 3/11 | 27.27% | 4/9 | 44.44% |  |

CMV, cytomegalovirus; Pre, Preoperative; Post, Postoperative; AVT, antivirals treatment; Y, yes, indicates the implementation of this treatment (antiviral treatment or glucocorticosteroid treatment); N, no, indicates the absence of this treatment (antiviral treatment or glucocorticosteroid treatment); w, week. Fisher exact probability method analyzed.
